# Insight into Romanian Wild-Grown *Heracleum sphondylium*: Development of a New Phytocarrier Based on Silver Nanoparticles with Antioxidant, Antimicrobial and Cytotoxicity Potential

**DOI:** 10.3390/antibiotics13090911

**Published:** 2024-09-23

**Authors:** Adina-Elena Segneanu, Gabriela Vlase, Titus Vlase, Ludovic Everard Bejenaru, George Dan Mogoşanu, Gabriela Buema, Dumitru-Daniel Herea, Maria Viorica Ciocîlteu, Cornelia Bejenaru

**Affiliations:** 1 Institute for Advanced Environmental Research, West University of Timişoara (ICAM–WUT), 4 Oituz Street, 300086 Timişoara, Timiş County, Romania; adina.segneanu@e-uvt.ro (A.-E.S.); gabriela.vlase@e-uvt.ro (G.V.); titus.vlase@e-uvt.ro (T.V.); 2 Research Center for Thermal Analyzes in Environmental Problems, West University of Timişoara, 16 Johann Heinrich Pestalozzi Street, 300115 Timişoara, Timiş County, Romania; 3 Department of Pharmacognosy & Phytotherapy, Faculty of Pharmacy, University of Medicine and Pharmacy of Craiova, 2 Petru Rareş Street, 200349 Craiova, Dolj County, Romania; george.mogosanu@umfcv.ro; 4 National Institute of Research and Development for Technical Physics, 47 Dimitrie Mangeron Avenue, 700050 Iaşi, Iaşi County, Romania; gbuema@phys-iasi.ro (G.B.); dherea@phys-iasi.ro (D.-D.H.); 5 Department of Analytical Chemistry, Faculty of Pharmacy, University of Medicine and Pharmacy of Craiova, 2 Petru Rareş Street, 200349 Craiova, Dolj County, Romania; maria.ciocilteu@umfcv.ro; 6 Department of Pharmaceutical Botany, Faculty of Pharmacy, University of Medicine and Pharmacy of Craiova, 2 Petru Rareş Street, 200349 Craiova, Dolj County, Romania; cornelia.bejenaru@umfcv.ro

**Keywords:** *Heracleum sphondylium*, silver nanoparticles, phytocomplex, secondary metabolites, antioxidant potential, antimicrobial screening, in vitro cytotoxicity

## Abstract

**Background**: *Heracleum sphondylium*, a medicinal plant used in Romanian ethnopharmacology, has been proven to have remarkable biological activity. The escalating concerns surrounding antimicrobial resistance led to a special attention being paid to new efficient antimicrobial agents based on medicinal plants and nanotechnology. We report the preparation of a novel, simple phytocarrier that harnesses the bioactive properties of *H. sphondylium* and silver nanoparticles (HS-Ag system). **Methods**: *H. sphondylium*’s low metabolic profile was determined through gas chromatography–mass spectrometry and electrospray ionization–quadrupole time-of-flight–mass spectrometry. The morphostructural properties of the innovative phytocarrier were analyzed by X-ray diffraction, Fourier-transform infrared spectroscopy, Raman spectroscopy, dynamic light scattering, scanning electron microscopy, and energy-dispersive X-ray spectroscopy. The antioxidant activity was evaluated using total phenolic content, ferric reducing antioxidant power, and 2,2-diphenyl-1-picrylhydrazyl (DPPH) in vitro assays. The antimicrobial activity screening against *Staphylococcus aureus*, *Bacillus subtilis*, *Pseudomonas aeruginosa*, and *Escherichia coli* was conducted using the agar well diffusion method. The 3-(4,5-Dimethylthiazol-2-yl)-2,5-diphenyltetrazolium bromide (MTT) assay estimated the in vitro potential cytotoxicity on normal human dermal fibroblasts (NHDF) and cervical cancer (HeLa) cells. **Results**: A total of 88 biomolecules were detected, such as terpenoids, flavonoids, phenolic acids, coumarins, phenylpropanoids, iridoids, amino acids, phytosterols, fatty acids. The HS-Ag phytocarrier heightened efficacy in suppressing the growth of all tested bacterial strains compared to *H. sphondylium* and exhibited a significant inhibition of HeLa cell viability. **Conclusions**: The new HS-Ag phytocarrier system holds promise for a wide range of medical applications. The data confirm the capacity to augment the pertinent theoretical understanding in the innovative field of antimicrobial agents.

## 1. Introduction

*Heracleum sphondylium* (*Apiaceae* family), commonly known as hogweed or cow parsnip, is widespread in Europe, parts of Asia, and northern Africa, and is present throughout Europe except for in the extreme north and some Mediterranean regions [1,2,3,4,5]. In Romania, *H. sphondylium*, known locally as *Brânca ursului*, is common nationwide in various forms, frequent from lowlands to mountainous regions, in thickets, hayfields, meadows, riparian zones, sparse forests, and rocky grasslands [4,6,7]. The species exhibits high variability, leading to many mentioned subspecies (nine in European flora, three in Romanian flora) [4,5,6,7].

*H. sphondylium* is a biennial or perennial species with a thick, branched rhizome. The aerial stem is well developed, reaching heights of up to (150–) 200 (–350) cm and a 4–20 mm diameter. The leaves are highly variable, ranging from simple, undivided, or merely lobed to pinnatisect leaves with 3–5(7) asymmetrical, diversely lobed segments; the axil of the stem leaves is slightly swollen, rough-pubescent, or glabrous. The inflorescences are large, with umbels up to 25 cm in diameter, with up to 40 unequal rays, and with few or without bracts. The flowers have variously colored petals (white, yellow, pink, purple, greenish, or blue) and are often slightly pubescent externally. The ovary is glabrous, pubescent, or hispid. The fruits are strongly flattened, ellipsoidal, obovate, or nearly round, emarginate, with winged lateral ribs forming a delineated margin around them. The plants bloom from June to September [4,5,6,7].

*H. sphondylium* is used as a nutritional source in many regions globally; the stems, leaves, and inflorescences are utilized to obtain numerous preparations; e.g., in Eastern Europe and Northeastern Asia, various soups are made using this plant [1,2].

*H. sphondylium* roots, stems, leaves, and inflorescences are employed in traditional medicine in countries where it grows spontaneously to treat digestive disorders such as flatulence, dyspepsia, diarrhea, and dysentery, as well as hypertension, epilepsy, menstrual problems, and for wound healing, due to its analgesic, sedative, anti-infective, antioxidant, anticonvulsant, vasorelaxant, antihypertensive, carminative, tonic, and aphrodisiac properties [8,9,10,11,12,13,14].

Recent studies addressing the chemical composition of *H. sphondylium* have demonstrated the presence of a complex mixture of furocoumarins (bergapten, isopimpinellin, heraclenin), essential oil, polyphenolic compounds, phytosterols, pentacyclic triterpenes, and fatty acids [1,2,3,14,15,16,17]. Numerous studies reported multiple therapeutic properties, such as antioxidant, vasorelaxant, antimicrobial, antiviral, anti-inflammatory, antidiabetic, neuroprotective, and antitumor [1,12,13,14]. Despite its great pharmacological potential, most research focuses on several phytochemical categories extracted from different parts of this plant [1,12,13,14]. In addition, there is limited research on Romanian wild-grown *H. sphondylium* addressing only essential oil and phenolic compounds [8,16].

Furthermore, the variations in secondary metabolites amount to a function of various abiotic and biotic factors, growth stage, and extraction technique parameters (temperature, solvent polarity, duration, pH, etc.), which dictate the herb’s chemical profile and biological activity [18,19,20,21,22]. Conversely, recent research on natural compounds reported that several molecules exhibit low bioavailability due to reduced chemical stability and limited adsorption [23,24,25].

Antimicrobial resistance and tolerance emerge as paramount health concerns with severe repercussions on the therapeutic strategy of infectious diseases [24]. Antibiotic abuse or misuse for human health and the agri-food sector contributed significantly to rendering existing antimicrobials ineffective and exacerbating antimicrobial resistance. Without urgent measures, the depletion of antimicrobial alternatives will lead to a rise in infections related to antibiotic-resistant pathogens. It is urgent to identify new targeted antimicrobial agents against pathogenic microorganisms while mitigating the progression of antimicrobial resistance. Consequently, various strategies to overcome these challenges have been developed [25,26,27].

On the other hand, the implementation of nanotechnology in the biomedical field led to the development of advanced materials based on numerous phytoconstituents with high antimicrobial, antiviral, neuroprotective, and antitumor activity, which allowed researchers not only to overcome these constraints, but also to achieve a significant improvement in the pharmacological activity, controlled release, and specificity while minimizing toxicity [24,25,26,27,28].

To this end, various nanoparticles (NPs), such as platinum, silver, gold, iron oxide, titanium dioxide, zinc, silica, and copper, have been reviewed for biomedical applications [29]. Among these, the silver nanoparticles (AgNPs) stood out due to their broad applicative potential from bioengineering to diagnosis, detection, gene and drug delivery, vaccines, and antimicrobial agents to wound and bone treatment [29,30,31]. Their extensive growth development is due to their outstanding size-related physicochemical (size, shape, surface plasmon resonance, surface charge, high surface-to-volume ratio, chemical stability, low reactivity) and biological (antimicrobial) properties [30,31]. In addition, AgNPs display a uniquely tailored hydrophilic–hydrophobic balance through simple functionalization with various molecules, and the capability to cross the blood–brain barrier ensures the opening of new possibilities in the design of drug delivery systems and new performant antimicrobial agents [30,31,32]. In that sense, research on developing engineered herbal formulation assembles using NPs represents a significant advancement in enhancing the biological properties of phytoconstituents and enabling specific targeting and localization on surfaces [29].

This study investigates the preparation of a new phytocarrier through *H. sphondylium* loading with AgNPs (HS-Ag system) encompassing the physical and chemical characteristics and in vitro evaluation of its antioxidant, antimicrobial, and cytotoxicity potential. To the best of our knowledge, the low metabolic profile of *H. sphondylium* grown wild in Romania is reported for the first time in this study.

## 2. Results

### 2.1. GC–MS Analysis of H. sphondylium Sample

**Figure 1:**
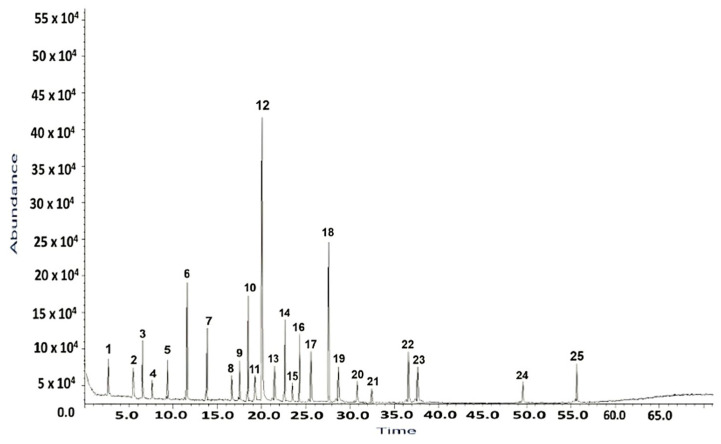
Total ion chromatogram of *H. sphondylium* sample.

The compounds separated using gas chromatography–mass spectrometry (GC–MS) are depicted in Figure 1 and detailed in Table 1.

The GC–MS analysis illustrates 25 compounds, constituting 82.38% of the total peak area in the *H. sphondylium* sample (Figure 1).

### 2.2. MS Analysis of H. sphondylium Sample

**Figure 2:**
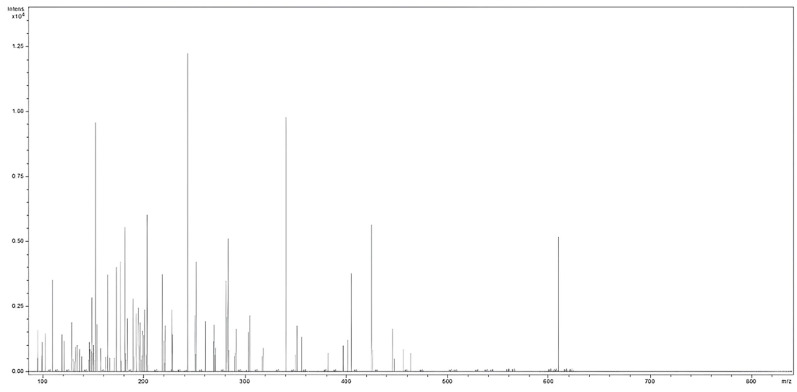
Mass spectrum of *H. sphondylium* sample.

The mass spectrum shown in Figure 2 indicates the presence of multiple biomolecules detected and assigned to various chemical categories from terpenes, fatty acids, flavonoids, phenolic acids, amino acids, hydrocarbons, organic acids, esters, sterols, coumarins, iridoids, phenylpropanoids, alcohols, and miscellaneous constituents. These results corroborate the data reported in the literature [1,2,8,10,14,15,16,17,47,48,49,50,51].

Table 2 highlights the phytochemicals identified via electrospray ionization–quadrupole time-of-flight–mass spectrometry (ESI–QTOF–MS) analysis.

### 2.3. Chemical Screening

**Figure 3:**
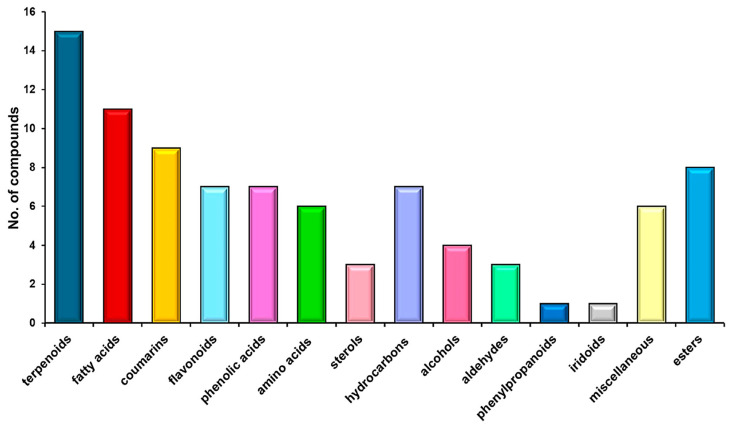
Phytochemical classification bar chart of *H. sphondylium* sample.

A total of 88 biomolecules identified through MS were appointed to various categories: terpenoids (17.04%), fatty acids (12.5%), coumarins (10.22%), flavonoids (7.95%), phenolic acids (7.95%), amino acids (6.81%), phytosterols (3.40%), esters (9.09%), hydrocarbons (7.95%), alcohols (4.54%), aldehydes (3.40%), phenylpropanoids (1.13%), iridoids (1.13%), and miscellaneous. Figure 3 shows the arrangement chart bar of phytochemicals from *H. sphondylium* according to the results of MS analysis (Table 2).

### 2.4. Key Aroma-Active Compounds Forming Different Flavor Characteristics

**Figure 4:**
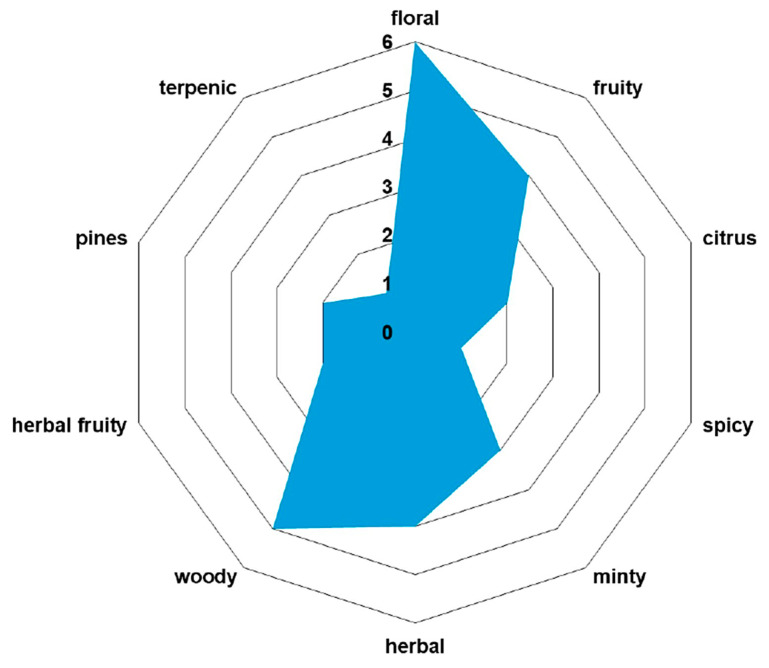
VOC odor profile compounds identified in *H. sphondylium* sample. VOC: volatile organic compound.

The volatile organic compound (VOC) odor profile of biomolecules identified in the *H. sphondylium* sample is presented in Table 3 and Figure 4.

### 2.5. Phytocarrier Engineered System

#### 2.5.1. FTIR Spectroscopy

**Figure 5:**
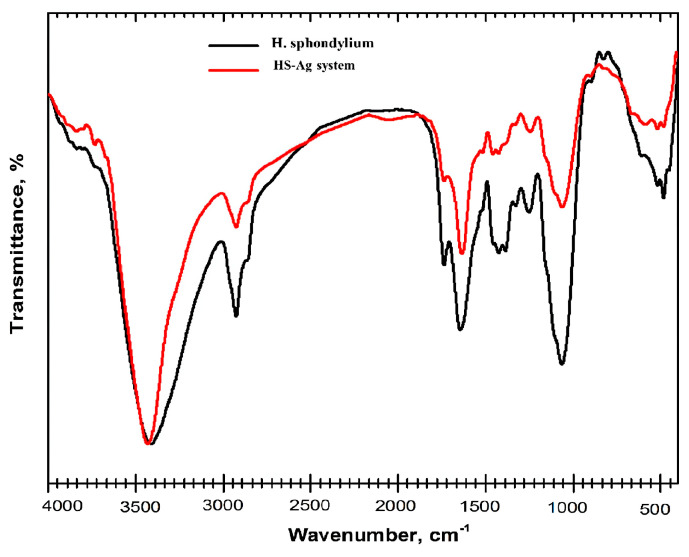
FTIR spectra of *H. sphondylium* sample and HS-Ag system. FTIR: Fourier-transform infrared; HS-Ag: *H. sphondylium*–silver nanoparticle system.

Fourier-transform infrared (FTIR) spectroscopy was utilized to examine the chemical interaction between AgNPs and phytoconstituents in plants, besides the formation of the phytocarrier system. Analysis of the *H. sphondylium* sample (Figure 5; Table 4) revealed the presence of various categories of biomolecules, including terpenoids, fatty acids, flavonoids, coumarins, phenolic acids, amino acids, phytosterols, aldehydes, esters, iridoids, and phenylpropanoids.

The FTIR spectrum of the HS-Ag system exhibits the characteristic vibrational bands of the *H. sphondylium* sample (Figure 5). These include peaks at approximately 2922 cm^−1^ corresponding to the asymmetric vibration of the CH_2_ groups from amino acids, at ~2848 cm^−1^ attributed to the symmetric vibration of the CH_2_ groups from fatty acids, and at ~1746 cm^−1^ attributed to the C=O stretch of terpenoids. Additionally, the spectra show a peak at ~1644 cm^−1^ assigned to the N–H stretch of amino acids, at ~1458 cm^−1^ attributed to the aromatic ring of phenolic acids, and at ~1242, 1060, and ~1016 cm^−1^ associated with the C–N vibration of amines. Furthermore, peaks at ~882 and ~814 cm^−1^ are assigned to C–O and C–H vibrations of aromatic rings, indicating the presence of AgNPs coated with sodium citrate [32].

Nonetheless, the following vibrational peaks at ~1632, 1389, 1114, and 675 cm^−1^, characteristic of AgNPs coated with the surfactant, exhibit observable shifts to higher wavenumbers (1642, 1392, 1118, and 681 cm^−1^) [32,60]. The spectral shifts observed indicate the interaction between AgNPs and the O–H, C=O, N–H, and C–O functional groups of the phytochemicals present in *H. sphondylium* sample. Notable changes in the herbal sample spectra are evident, particularly in the vibrational absorption at around 3407, 1412, and 1380 cm^−1^ (O–H), besides 1292, 1150, and 1060 cm^−1^ (C–O). These shifts to higher wavenumbers suggest the involvement of these functional groups in binding the AgNPs, possibly through hydrogen bonding. Furthermore, the distinct sharpening observed in the O–H and N–H stretching regions shows distinct sharpening support evidence for HS-Ag system preparation.

#### 2.5.2. XRD Analysis

**Figure 6:**
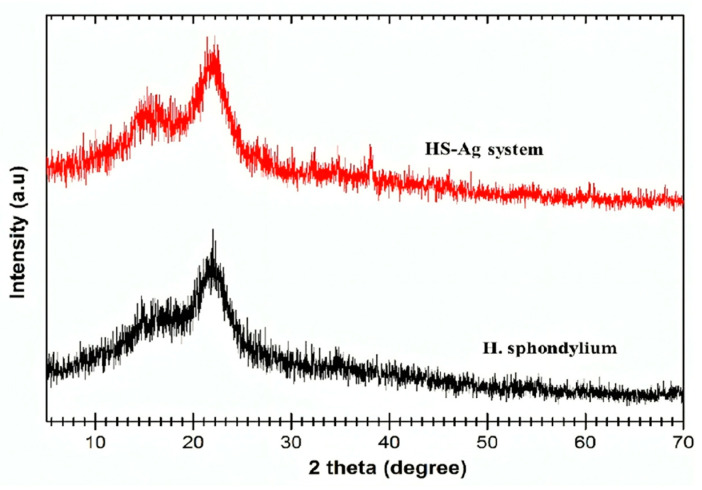
Powder XRD patterns of *H. sphondylium* sample and HS-Ag system. HS-Ag: *H. sphondylium*–silver nanoparticle system; XRD: X-ray diffraction.

The X-ray diffraction (XRD) patterns of *H. sphondylium* sample and HS-Ag system are shown in Figure 6.

The HS-Ag system XRD pattern displays the diffraction peaks of *H. sphondylium* biomolecules (at 2θ: 15.78° and 22.21°) and AgNPs (at 2θ: 27.87°, 38.15°, 64.4°, and 78.5°) [32,61,62].

Notably, the distinctive peaks of phytoconstituents are shifted to lower angles, indicating the incorporation of AgNPs into the herbal matrix. The interaction between AgNPs and the herbal matrix induces structural modifications, as evidenced by the discernible shift in XRD peak positions, reflecting the influential impact of metallic NPs on the herbal matrix amorphous structure.

#### 2.5.3. SEM and EDX Analysis

**Figure 7:**
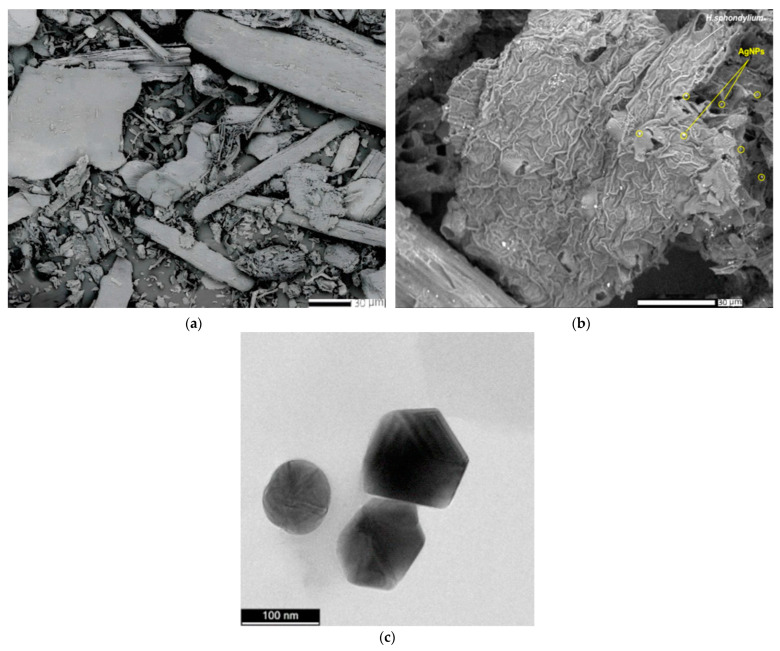
SEM images of *H. sphondylium* sample (**a**) and HS-Ag system (**b**). HR-TEM image of AgNPs (**c**). HR-TEM: high-resolution transmission electron microscopy; HS-Ag: *H. sphondylium*–silver nanoparticle (AgNP) system; SEM: scanning electron microscopy.

Figure 7a,b presents the scanning electron microscopy (SEM) images for the *H. sphondylium* sample and the HS-Ag system.

The SEM image of the *H. sphondylium* sample (Figure 7a) revealed a complex structure comprising particles of various shapes and sizes. The HS-Ag system (Figure 7b) demonstrated a modification in the morphology of the *H. sphondylium* sample, with numerous nanosized spherical Ag particles (~19 nm) visibly present on the surface and within the pores of the herbal matrix particles. In the SEM image shown in Figure 7b, a few of the AgNPs from the HS-Ag system have been highlighted by encircling them in yellow to emphasize the loading of the AgNPs onto the surface and within the pores of the herbal matrix.

The morphology, shape, and dimensions of the synthesized AgNPs were thoroughly examined using high-resolution transmission electron microscopy (HR-TEM). The analysis revealed that the AgNPs exhibit a spherical morphology, with average sizes ranging from 20 to 40 nm, as depicted in Figure 7c.

**Figure 8:**
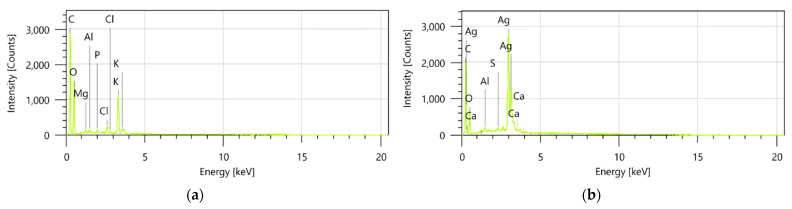
EDX composition of *H. sphondylium* sample (**a**) and HS-Ag system (**b**). EDX: energy-dispersive X-ray; HS-Ag: *H. sphondylium*–silver nanoparticle system.

Moreover, the energy-dispersive X-ray (EDX) spectra of the HS-Ag system showed characteristic peaks corresponding to both *H. sphondylium* sample and AgNPs, as depicted in Figure 8a,b, confirming the successful preparation of the newly engineered phytocarrier.

#### 2.5.4. DLS Analysis

**Figure 9:**
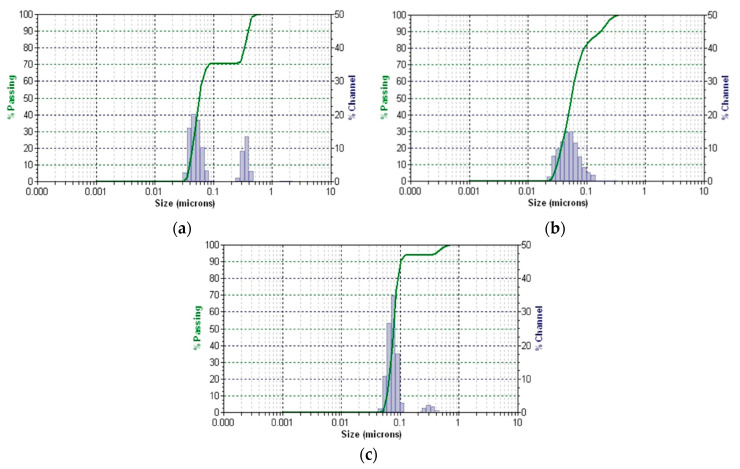
DLS patterns of *H. sphondylium* sample (**a**), citrate-coated AgNPs (**b**) and HS-Ag system (**c**). DLS: dynamic light scattering; HS-Ag: *H. sphondylium*–silver nanoparticle (AgNP) system.

The study results on the stability and dynamics of herbal matrix particles, citrate-coated AgNPs and a new system obtained by the dynamic light scattering (DLS) method are shown in Figure 9a–c.

The hydrodynamic diameter of the AgNPs obtained, as determined by DLS, was measured to be 33 ± 4 nm, with a polydispersity index (PDI) of 0.15. This finding is consistent with the results from XRD and SEM, as the size determined using DLS reflects the hydrodynamic size rather than the physical size.

The DLS profile of the *H. sphondylium* sample and HS-Ag system displays two distinct peaks within a narrow range, indicating the presence of two particle populations for each sample. The sizes are 0.049 μm and 0.36 μm, with a PDI of 0.17 and 0.18 for the herbal matrix particles, and 0.039 μm and 0.26 μm, with a PDI of 0.26 and 0.29 for the HS-Ag system. The PDI values (PDI lower than 0.3) confirm a narrow size distribution of the NPs across all measured fractions. The observed visual stability of the suspensions is supported by the low PDI value of the samples in combination with their nanometric size.

Conversely, the narrow range of the peaks indicates high stability [63]. Additionally, the decrease in particle size in the HS-AgNPs system results in a higher surface area, leading to faster and more effective dissolution than in the *H. sphondylium* sample.

### 2.6. Total Phenolic Content and Screening of Antioxidant Potential

**Figure 10:**
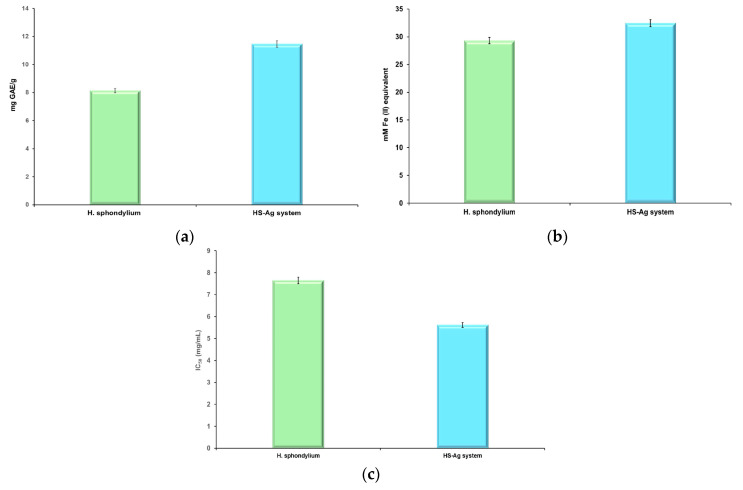
Graphic representation of TPC (**a**), FRAP (**b**), and DPPH (**c**) assay outcomes. DPPH: 2,2-Diphenyl-1-picrylhydrazyl; FRAP: ferric reducing antioxidant power; GAE: gallic acid equivalents; HS-Ag: *H. sphondylium*–silver nanoparticle system; IC_50_: half maximal inhibitory concentration; TPC: total phenolic content.

To comprehensively assess the antioxidant capacity, two specific in vitro assays—ferric reducing antioxidant power (FRAP) and 2,2-diphenyl-1-picrylhydrazyl (DPPH)—were selected. In addition, the total phenolic content (TPC) assay was used to evaluate the total phenolic compounds in the herbal product and the HS-Ag system. The results are illustrated in Figure 10a–c and Table 5.

The findings from the TPC assay indicate a substantial rise in phenolic content (40.91%) in the HS-Ag system compared to *H. sphondylium*, which is attributed to the catalytic properties of AgNPs [64]. The FRAP assay data also demonstrate a moderate increase (10.67%) in reducing power for the HS-Ag system over *H. sphondylium*. Furthermore, the DPPH radical scavenging assay results reveal a significant decrease (26.53%) in the half maximal inhibitory concentration (IC_50_) value for scavenging activity associated with a higher antioxidant activity.

### 2.7. Antimicrobial Screening

The screening of antimicrobial activity against selected pathogenic microorganisms was tested in this study, specifically against *Staphylococcus aureus* (Gram-positive), *Bacillus subtilis* (Gram-positive), *Pseudomonas aeruginosa* (Gram-negative), and *Escherichia coli* (Gram-negative), using the agar well diffusion method. *H. sphondylium* and a newly prepared HS-Ag system were evaluated for their antibacterial activity by measuring the diameter of inhibition zones (IZs) and comparing the results with positive (Gentamicin) and negative (dimethyl sulfoxide—DMSO) controls. The data presented in Table 6 indicate that both samples (*H. sphondylium* and HS-Ag system) exhibited strong antibacterial activity against all tested pathogenic microorganisms.

Notably, even at the lowest concentration tested (100 μg/mL), the herbal sample, citrate-coated AgNPs and the HS-Ag system showed significantly larger IZ diameters compared to the positive control (Gentamicin) against both Gram-positive bacteria strains (*S. aureus* and *B. subtilis*). However, for the Gram-negative bacteria strains, the IZs obtained for the lowest concentration of the herbal sample (100 μg/mL) were lower than Gentamicin (18.67% against *P. aeruginosa* and 20.69% against *E. coli*). Regarding the antimicrobial activity of citrate-coated AgNPs against Gram-negative strains, it was observed that the highest concentration of AgNPs (200 μg/mL) exhibited a similar IZ diameter to Gentamicin against *P. aeruginosa*. In contrast, even at a concentration of 150 μg/mL, citrate-coated AgNPs showed a similar IZ diameter against *E. coli*. Furthermore, at higher concentrations of citrate-coated AgNPs (175 and 200 μg/mL), IZ diameters were larger than Gentamicin against *E. coli*. On the other hand, the HS-Ag system’s lower concentration (100 μg/mL) displayed a slightly larger IZ diameter than Gentamicin against *P. aeruginosa* (16.65%). Meanwhile, the antibacterial IZs against *E. coli* obtained for the same concentration of the HS-Ag system (100 μg/mL) were almost like Gentamicin. Finally, the highest concentrations of all samples, the herbal sample, citrate-coated AgNPs and the HS-Ag system (200 μg/mL) demonstrated the largest IZ diameters against all tested bacterial strains. Additionally, the HS-Ag system was more effective at inhibiting the growth of all tested bacterial strains at all concentrations than *H. sphondylium*.

To confirm the antibacterial efficacy of samples (*H. sphondylium*, citrate-coated AgNPs and the newly formulated HS-Ag system), the minimum inhibitory concentration (MIC) and minimum bactericidal concentration (MBC) were determined against all bacterial strains. The results are illustrated in Table 7.

All samples demonstrated significant antimicrobial activity in the MIC and MBC assays. The MIC value of *H. sphondylium* sample varied from 0.22 ± 0.07 to 0.98 ± 0.11 μg/mL, and from 0.13 ± 0.04 to 0.67 ± 0.17 μg/mL for citrate-coated AgNPs, while for the HS-Ag system, it ranged from 0.12 ± 0.03 to 0.52 ± 0.07 μg/mL. Correspondingly, the MBC values for all investigated samples aligned closely with the MIC values. These results demonstrated a superior antibacterial effect of the HS-Ag system compared to herbal and citrate-coated AgNPs samples across all bacterial strains tested. It is worth noting that the MIC and MBC values for all samples are lower than those of Gentamicin (positive control). The bacterial growth was absent in the negative control, which only contained nutrient broth.

### 2.8. Cell Viability Assay

**Figure 11:**
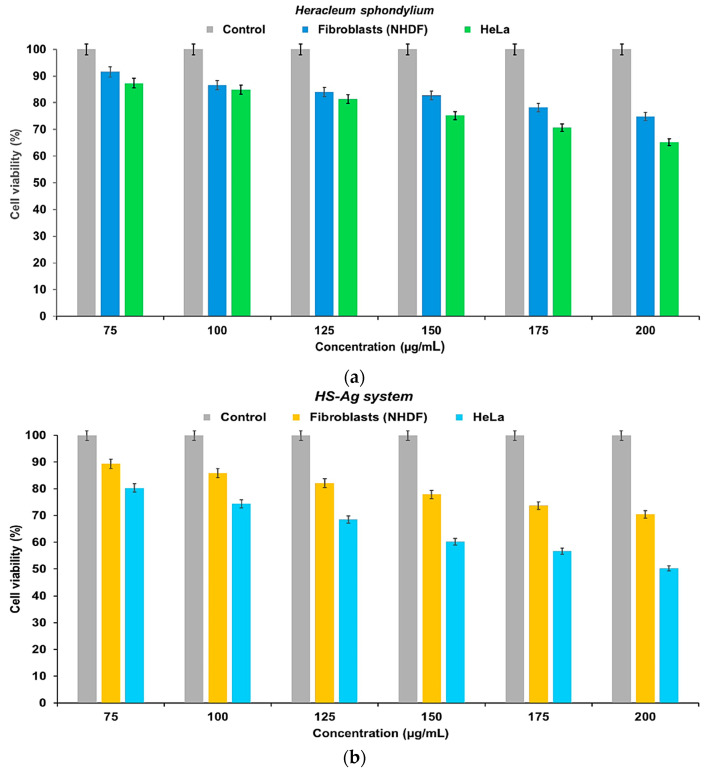
Viability of NHDF and HeLa cells, 24 h after co-incubation with different concentrations of *H. sphondylium* sample (**a**) and HS-Ag system (**b**). Positive control wells contained untreated cells, MTT solution, and DMSO. Data are presented as mean ± SEM of three independent readings (*n* = 3). DMSO: Dimethyl sulfoxide; HeLa: Henrietta Lacks; HS-Ag: *H. sphondylium*–silver; MTT: 3-(4,5-Dimethylthiazol-2-yl)-2,5-diphenyltetrazolium bromide; NHDF: normal human dermal fibroblasts; SEM: Standard error of the mean.

Figure 11a,b illustrates the results of cell viability testing using the 3-(4,5-dimethylthiazol-2-yl)-2,5-diphenyltetrazolium bromide (MTT) assay on *H. sphondylium* and HS-Ag system samples at various concentrations (75, 100, 125, 150, 175, and 200 μg/mL).

The data suggest that lower concentrations of *H. sphondylium* correspond to higher cell viability, indicating a less toxic effect on the normal human dermal fibroblasts (NHDF) cell line. A constant, slight decrease in cell viability was observed within the 75–150 μg/mL concentration range. At higher concentrations of 175 and 200 μg/mL, a more significant decrease in cell viability occurred, but it remained above 74% (Figure 11a).

In the case of the cervical cancer (Henrietta Lacks—HeLa) cell line, there was a consistent decrease in cell viability as the concentration of the herbal extract increased. The most notable impact occurred at higher concentrations (175 and 200 μg/mL) (Figure 11b).

Similarly, in the case of the HS-Ag system, the outcomes of the MTT assay indicated that cell viability was dose-dependent. Thus, the NHDF cells displayed a continuous decrease in cell viability when the HS-Ag system concentration increased. Notably, at 200 μg/mL, the maximum concentration of the HS-Ag system corresponded to the lower cell viability value (70.46 μg/mL) but remained above the standard value (Figure 11a).

However, the HS-Ag system had a notably more pronounced negative impact on the HeLa tumor cell line, with an inversely proportional relationship between concentration and cell viability. Specifically, the maximum effect of 50.26% was observed at 200 μg/mL of the HS-Ag system (Figure 11b).

**Figure 12:**
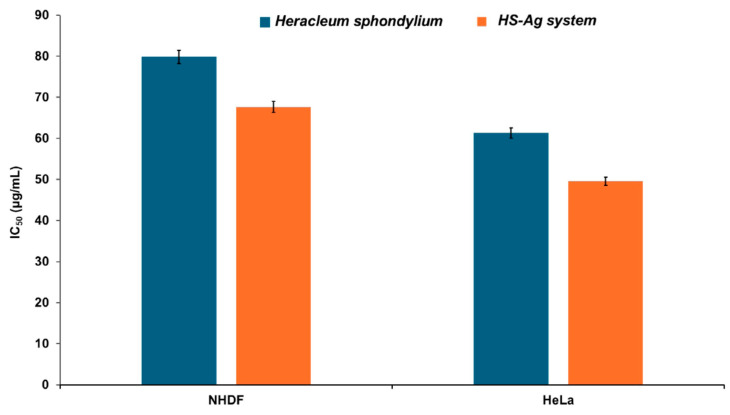
In vitro cytotoxicity of HS-Ag system vs. *H. sphondylium*, as a function of concentration against NHDF and HeLa cell lines (after 24 h). Data are presented as mean ± SEM of three independent readings (*n* = 3). HeLa: Henrietta Lacks; HS-Ag: *H. sphondylium*–silver nanoparticle system; IC_50_: half maximal inhibitory concentration; NHDF: normal human dermal fibroblasts; SEM: standard error of the mean.

The IC_50_ values of in vitro cytotoxicity calculated for *H. sphondylium* are higher than those for the HS-Ag system, as illustrated in Figure 12.

Thus, for NHDF cells, the IC_50_ values of *H. sphondylium* and the HS-Ag system were 79.82 ± 0.023 and 67.65 ± 0.019 μg/mL, respectively. For HeLa cells, the IC_50_ values of *H. sphondylium* and the HS-Ag system were 61.31 ± 0.078 and 49.54 ± 0.064 μg/mL, respectively. The data suggest that the HS-Ag system exhibits higher cytotoxicity than *H. sphondylium* against tumor cells (19.18%).

## 3. Discussion

*H. sphondylium*, a renowned medicinal plant with well-established therapeutic properties in Romanian ethnomedicine, has gained recent attention due to its remarkable biological activity. The escalating concerns surrounding antimicrobial resistance led to a critical reevaluation of current therapeutic strategies for infectious diseases. Recent research focuses on the new selective targeting strategies for innovative antimicrobial agents. Special attention is paid to new efficient antibiotics based on medicinal plants and nanotechnology.

### 3.1. Screening and Classification of the Different Metabolites of H. sphondylium

Concerning the chemical composition of *H. sphondylium*, a total of 88 biomolecules were detected through GC–MS and ESI–QTOF–MS, encompassing a diverse array of categories, mainly terpenoids, coumarins, flavonoids, phenolic acids, amino acids, fatty acids, phytosterols, phenylpropanoids, and iridoids.

Terpenoids represent over 17% of the total *H. sphondylium* phytoconstituents (Figure 3). The therapeutic properties of terpenoids are multiple, including anti-inflammatory, antimicrobial, antiviral, antitumor, analgesic, cardioprotective, antispastic, antihyperglycemic, and immunomodulatory [65].

Coumarins are the third class of metabolites, representing over 10% of the phytochemicals from the hogweed sample (Figure 3). Research has reported that these secondary metabolites possess high antioxidant, antiviral, anti-inflammatory, antitumor, neuroprotective, anticoagulant, anticonvulsant, cardioprotective, antihypertensive, immunomodulatory, and antidiabetic properties [54,66].

Flavonoids, which comprise approximately 8% of the *H. sphondylium* sample (Table 2; Figure 3), are metabolites with outstanding biological activities: antimicrobial, antioxidant, cardioprotective, antiviral, neuroprotective, and antitumor [67].

Phenolic acids represent a significant class of phytochemicals identified in the composition of the *H. sphondylium* sample (Table 2; Figure 3). Research showed that these metabolites exhibit anti-inflammatory, antibacterial, antioxidant, antidiabetic, anti-allergic, antitumor, cardioprotective, and neuroprotective properties [68,69].

Amino acids are another category of phytochemicals encompassing over 83% of non-essential amino acids (glycine, alanine, serine, aspartic acid, glutamic acid) (Table 2). About 50% of these compounds (glycine, alanine, glutamic acid) exert antiproliferative and immunomodulatory activity. Over 33% (serine and threonine) act as anti-inflammatory agents. In addition, studies report the beneficial effect of aspartic acid on neurological and psychiatric diseases [70,71].

Fatty acids comprise 12.5% of total phytochemicals from the *H. sphondylium* sample, with about 72% saturated fatty acids (capric, stearic, behenic, lauric, myristic, margaric, arachidic, and palmitic acids), two monosaturated fatty acids (oleic and palmitoleic acids) and one ω-6 acid (linoleic acid) (Table 2). These compounds possess anti-inflammatory, antioxidant, antimicrobial, neuroprotective, and cardioprotective properties [72].

Phytosterols represent over 3% of the total phytochemicals (Table 2) and act as antioxidant, neuroprotective and cardioprotective, anti-inflammatory, antitumor, and immunomodulatory agents [73].

The phenylpropanoid estragole (Table 2) displays antibacterial, antiviral, antioxidant, anti-inflammatory, and immunomodulatory activity [74].

Iridoid compound loganic acid (Table 2) possesses neuroprotective, anti-inflammatory, antioxidant, and antiadipogenic effects [75].

### 3.2. New Phytocarrier System with Antioxidant, Antimicrobial and Cytotoxicity Potential

The utilization of nanotechnology and the advancement of engineered delivery systems employing metallic NPs circumvent the in vitro deficiencies, particularly stability and reduced adsorption, associated with certain phytoconstituents possessing heightened biological activity. These tailored systems promote targeted activity, prolonged drug release, reduced drug doses, and lowered toxicity. Additionally, they can improve the therapeutic effects by combining the actions of the herbal compounds and the metallic NPs [22,23,76]. As a result, a new delivery system based on AgNPs was developed from *H. sphondylium*.

Multiple assays provide a thorough and precise evaluation of the antioxidant potential of herbal products. In vitro tests are particularly valuable for assessing the antioxidant activity of samples containing complex compositions of biomolecules. The antioxidant activity of *H. sphondylium* is intricately linked to the highly active phytoconstituents.

The biological activity of AgNPs, particularly their antibacterial activity, is closely linked to the size and shape of the particles, as well as their high surface-to-volume ratio and concentration [32].

Conversely, the antioxidant potential within the HS-Ag system is derived from the phytochemicals and AgNPs conjugate effect. The results suggest that in the HS-Ag system, AgNPs, in conjunction with the phytoconstituents, could act as hydrogen donors, reducing agents, and singlet oxygen quenchers [77].

The results suggest that the antimicrobial efficacy of all samples is dose-dependent, consistent with the existing literature [32,78].

Gram-positive bacterial strains (*S. aureus* and *B. subtilis*) exhibited a greater sensitivity to both *H. sphondylium*, citrate-coated AgNPs and HS-Ag system samples compared to Gram-negative bacteria (*P. aeruginosa* and *E. coli*), possibly attributed to morphological variances within these distinct microorganism categories. Additionally, the outer membrane features of Gram-negative bacteria may act as a barrier against various compounds [79].

The antimicrobial activity of the *H. sphondylium* sample can be attributed to its complex mixture of phytoconstituents renowned for their antimicrobial properties, encompassing flavonoids, terpenoids, phenolic acids, fatty acids, and phenylpropanoids (estragole, anethole, myristicin) [80,81].

Notably, phenolic acids impact the bacterial membrane and cytoplasmic levels, while flavonoids act on the membrane level and inhibit deoxyribonucleic (DNA) and ribonucleic (RNA) synthesis [69]. Furthermore, terpenoids restrict bacterial respiration and oxidative phosphorylation [82,83].

Conversely, the antimicrobial activity of the HS-Ag system may be ascribed to the synergistic biological mechanism of phytochemicals and AgNPs. While the biological mechanism of AgNPs remains elusive, numerous studies have reported that AgNPs disrupt membrane interactions, deactivate proteins through Ag^+^ interaction and adversely impact bacterial DNA [32]. Furthermore, the antibacterial properties of AgNPs depend on other variables such as particle shape and concentration.

The lower values of the MIC and MBC are associated with the most efficient antimicrobial effect [79]. The *H. sphondylium* sample displayed the lowest MIC values against *B. subtilis*, followed by *S. aureus*, *E. coli*, and *P. aeruginosa*. The bacterial susceptibility diversity could be associated either with their resistance or the sample composition, specifically with the conjugate antimicrobial effect of different categories of phytoconstituents in the case of *H. sphondylium*, multiplied by the presence of AgNPs in the HS-Ag system [79]. Furthermore, all bacteria employed in this study are associated with various infections. Research has demonstrated that Gram-negative microorganisms are reservoirs for hospital-acquired infections, and there is a growing concern regarding drug-resistant infections attributable to Gram-negative bacteria [84]. Hence, the findings from this study advocate the potential utilization of the newly formulated HS-Ag system as an antimicrobial agent.

In vitro cytotoxicity assays are commonly employed to assess the potential toxicity of a specific compound on cell culture models. These assays ascertain the impact of the compound on cell viability, growth, morphology, and metabolism, as well as its ability to impede cell viability, cell growth, and proliferation, offering insights into its cytotoxicity as an initial step in bioavailability assessment. Among the various methods available, colorimetric assays, particularly the MTT assay, are widely utilized, considering their cost-effectiveness in vitro cell viability assessment [85,86,87]. The findings suggest that the herbal extract and the newly prepared engineered phytocarrier are not toxic to the NHDF cell line [87]. In the case of the cervical cancer (HeLa) cell line, a significant decrease in cell viability as the concentration of the herbal extract increased (175 and 200 μg/mL) was highlighted. Also, the results support the existing reported data [1]. Moreover, the HS-Ag system exhibited higher cytotoxicity than *H. sphondylium* against the tumor cell line. This finding could be attributed to the synergistic effects of phytoconstituents and the ability of AgNPs to facilitate the generation of reactive oxygen species (ROS) [88].

## 4. Materials and Methods

### 4.1. Chemicals and Reagents

All used reagents were of analytical grade. Ethanol, methanol, dichloromethane, chloroform, sodium carbonate, gallic acid, DPPH, acetate buffer solution (pH 4–7), FRAP assay kit (MAK369-1KT), and DMSO were acquired from Sigma Aldrich (München, Germany) and used without further purification. The MTT kit was obtained from AAT Bioquest (Pleasanton, CA, USA). Ultrapure water was used in all experiments.

### 4.2. Cell Lines

NHDF and HeLa cell lines were purchased from the American Type Culture Collection (ATCC; Manassas, VA, USA). Both cell lines were cultivated at 37 °C, in Dulbecco’s Modified Eagle’s Medium (DMEM; Gibco, Life Technologies, Leicestershire, UK), supplemented with 10% fetal bovine serum (FBS), and 1% antibiotic antimycotic solution (Sigma Aldrich).

### 4.3. Bacterial Strains

*S. aureus* (ATCC 29213), *B. subtilis* (ATCC 9372), *P. aeruginosa* (ATCC 27853), and *E. coli* (ATCC 25922) were purchased from the ATCC (Manassas, VA, USA).

### 4.4. Plant Material

The *H. sphondylium* samples (whole plant—stems of 165 cm in height, leaves, flowers of 25 cm diameter, and roots) were collected in June 2022 from the area of Timiş County, in Western Romania (geographic coordinates 45°43′02″ N, 21°19′31″ E) and taxonomically authenticated at the West University of Timişoara. Voucher specimens (HERA-SPD-2022-0806) were deposited at the Department of Pharmaceutical Botany, Faculty of Pharmacy, University of Medicine and Pharmacy of Craiova, Romania.

### 4.5. Preparation of AgNPs

AgNPs were prepared according to a procedure described in our previous paper [32].

### 4.6. Plant Sample Preparation for Chemical Screening

The freeze-dried plant samples (whole plant) were milled using a planetary Fritsch Pulverisette mill (Idar-Oberstein, Germany), at 720 rpm for 12 min at 24 °C, and then sieved through American Society for Testing Materials (ASTM) standard test sieve series to obtain particles of 0.25–0.30 mm range. The vegetal material was subjected to sonication extraction (Elmasonic, Singen, Germany) for 50 min at 45 °C and 65 Hz and dissolved in methanol (20 mL). All extracts were prepared in triplicate.

### 4.7. GC–MS Analysis

GC analysis was performed using the GCMS-QP2020NX Shimadzu equipment (Kyoto, Japan) provided with a ZB-5MS capillary column (30 m length, 0.25 mm inner diameter, 0.25 μm film thickness) from Agilent Technologies (Santa Clara, CA, USA). Helium was used as the carrier gas at a flow rate of 1 mL/min.

#### 4.7.1. GC–MS Separation

The oven temperature program was initiated at 50 °C, held for 2 min, and subsequently ascended to 300 °C at a rate of 5 °C per minute, where it was maintained for 4 min. The injector’s temperature was registered at 280 °C, while the interface temperature at 225 °C. Compound mass was measured at an ionization energy of 70 eV, commencing after a 2 min solvent delay. The mass spectrometer source and MS Quad were maintained at 225 °C and 160 °C, respectively. The compounds’ identification was accomplished based on their mass spectra, compared with the USA National Institute of Standards and Technology (NIST) 2.0 software (NIST, Gaithersburg, MD, USA) database, and supplemented with a literature review.

#### 4.7.2. Mass Spectrometry

The MS experiments were carried out using an ESI–QTOF–MS analysis system (Bruker Daltonics, Bremen, Germany). The mass spectra were acquired in the positive ion mode over a mass range of 100 to 3000 *m*/*z*, with a scan speed of 2.0 scans per second, a collision energy ranging from 25 to 85 eV, and a source block temperature set at 85 °C. The identification of phytoconstituents relied on the standard library NIST/National Bureau of Standards (NBS)-3 (NIST, Gaithersburg, MD, USA) and was supplemented with a literature review. The obtained mass spectra values and the identified secondary metabolites are shown in Table 2.

### 4.8. Phytocarrier System Preparation (HS-Ag System)

The HS-Ag system was prepared by mixing *H. sphondylium* (solid herb samples prepared as previously described) with an AgNPs solution in a 1:3 mass ratio. The obtained mixture was subjected to ultrasonic mixing for 20 min at 40 °C, and then filtered (F185 mm filter paper) and dried in an oven at 40 °C for 6 h. Each experiment was carried out in triplicate.

### 4.9. Characterization of HS-Ag System

#### 4.9.1. FTIR Spectroscopy

Data collection was conducted after 30 recordings at a resolution of 4 cm^–1^, in the range of 4000–400 cm^–1^, on Shimadzu AIM-9000 spectrometer with attenuated total reflectance (ATR) devices (Shimadzu, Tokyo, Japan). The assignment of wavelengths was based on a literature review.

#### 4.9.2. XRD Spectroscopy

The X-ray powder diffraction (XRD) was carried out on a Bruker AXS D8-Advance X-ray diffractometer (Bruker AXS GmbH, Karlsruhe, Germany), CuKα radiation, *k* 0.1541 nm, equipped with a rotating sample stage, Anton Paar TTK low-temperature cell (−180 °C to 450 °C), high-vacuum, inert atmosphere, and relative humidity control, Anton Paar TTK high-temperature cell (up to 1600 °C). The XRD patterns were compared with those from the International Centre for Diffraction Data (ICDD) Powder Diffraction Database (ICDD file 04-015-9120). The average crystallite size and the phase content were determined using the whole-pattern profile-fitting (WPPF) method.

#### 4.9.3. SEM Analysis

SEM micrographs were captured utilizing an SEM–energy-dispersive X-ray spectroscopy (EDS) system (Quanta Inspect F50; FEI-Philips, Eindhoven, The Netherlands) equipped with a field-emission gun (FEG), providing a resolution of 1.2 nm. Additionally, the system incorporates an EDX spectrometer, with an MnK resolution of 133 eV.

#### 4.9.4. DLS Particle Size Distribution Analysis

DLS analysis was conducted on a Microtrac/Nanotrac 252 (Montgomeryville, PA, USA). Each sample was analyzed in triplicate at room temperature (22 °C) at a scattering angle of 172°.

### 4.10. Assessment of the Total Phenolic Content and Antioxidant Activity

The assessment of the total phenolic compounds in the herbal product and the HS-Ag system was carried out by TPC (Folin–Ciocalteu assay). The antioxidant activity of the *H. sphondylium* sample and of the HS-Ag system was evaluated using two different methods: FRAP and DPPH. All experiments for antioxidant activity screening were performed in triplicate.

#### 4.10.1. Sample Preparation

Separately, 0.22 g of the *H. sphondylium* sample and 0.22 g of the HS-Ag system were added to 6 mL of 70% ethanol. Following a 10 h stirring period at room temperature (23 °C), the mixtures were centrifuged at 5000 rpm for 8 min. The resulting supernatant was collected for further evaluation of the antioxidant potential of each sample.

#### 4.10.2. Determination of TPC

The TPC of the *H. sphondylium* and HS-Ag system samples prepared as stated above (*vide supra*) was determined spectrophotometrically (FLUOstar Optima UV-Vis spectrometer; BMG Labtech, Offenburg, Germany) according to the Folin–Ciocalteu procedure adapted from our earlier publication [64]. The results were expressed in gallic acid equivalents (mg GAE/g sample). Sample concentrations were calculated based on the linear Equation (1) obtained from the standard curve and the correlation coefficient (R^2^ = 0.9997):

*y* = 0.0021*x* + 0.1634 (1)

#### 4.10.3. FRAP Assay

The FRAP antioxidant activity of the *H. sphondylium* and HS-Ag system samples was determined spectrophotometrically (FLUOstar Optima UV-Vis spectrometer; BMG Labtech) at 595 nm, using a FRAP Assay Kit, according to the procedure described in our earlier publication [36]. The results were expressed in mM Fe^2+^, calculated according to Equation (2):

(2) $$FRAP=\frac{mM{Fe}^{2+}\times F_{D}}{V}$$

where *FRAP*: ferric reducing antioxidant power; *mMFe*^2+^: iron ions (Fe^2+^) amount generated from the calibration curve of each sample (mM); *F_D_*: dilution factor; *V*: volume of each sample (μL).

#### 4.10.4. DPPH Radical Scavenging Assay

The DPPH radical scavenging activity of the *H. sphondylium* and HS-Ag system samples was performed according to the procedure described in our earlier publication [64]. The absorbance (*A*) was recorded at 520 nm (FLUOstar Optima UV-Vis spectrometer; BMG Labtech). The IC_50_ values (μg/mL) were determined from the inhibition percentage, *Inh*(%), from the calibration curve generated for each sample, according to Equation (3):

(3) $$Inh(\%)=\frac{\left(A_{0}-A_{1}\right)}{A_{0}}\times 10$$

### 4.11. Antimicrobial Test

Agar well diffusion assay, MICs, and MBCs were conducted to evaluate the antimicrobial activity of *H. sphondylium* and HS-Ag system.

MICs and MBCs were determined using the microbroth dilution method (Mueller–Hinton medium). MIC was considered the lowest compound concentration that inhibits bacterial growth, while MBC represents the lowest concentration at which no visible bacterial growth occurs after 14 h incubation. The microorganism growth inhibition was evaluated as the optical density at 600 nm using a T90+ UV–Vis spectrophotometer (PG Instruments, Lutterworth, UK) [89].

Nutrient agar and nutrient broth were prepared according to the manufacturer’s instructions and autoclaved at 120 °C for 20 min. The final concentration of microorganisms was adjusted to 0.5 McFarland Standard (1.5 × 10^8^ CFU/mL; CFU: Colony-forming unit). Each assay was performed in triplicate [89].

The diluted sections of five concentrations (100, 125, 150, 175, and 200 μg/mL) were prepared using 25% DMSO [89].

The antimicrobial potential of *H. sphondylium* and the HS-Ag system was evaluated using the agar well diffusion method according to the experimental procedure adapted from the literature [79,90,91].

The bacterial strains were initially cultured on a nutrient substrate and then inoculated for 24 h. Circular wells were created using a sterile glass capillary (5 mm). The bacterial strains (4–6 h) were streaked onto the nutrient agar using a sterile swab, and this process was repeated three times, with the plate rotated between each streaking. Next, 1 mL from each sample (*H. sphondylium* and HS-Ag system) concentration was introduced into the designated wells. The plates were then placed in an incubator at 37 °C for 24 h and later analyzed to determine the IZs. DMSO served as the negative control, while Gentamicin (100 μg/mL) was used as the positive control. The diameter (mm) of the IZs around the discs was measured using a ruler to determine the extent of bacterial growth inhibition. Each assay was performed in triplicate [79,90,91].

### 4.12. Cell Culture Procedure

#### 4.12.1. Cell Culture and Treatment

The cell lines utilized in this study included NHDF and HeLa cells (ATCC; Manassas, VA, USA). The cells were cultured at 37 °C under 5% carbon dioxide (CO_2_) and 100% humidity in DMEM supplemented with FBS and 1% antibiotic antimycotic solution. After seeding the cells at a density of 4 × 10^3^ cells/well in 96-well plates, they were allowed to reach 90% confluency over 24 h. Subsequently, the culture medium was replaced with a fresh medium containing varying concentrations (75, 100, 125, 150, 175, and 200 μg/mL) of *H. sphondylium* and the HS-Ag system. The cells were then cultured for an additional 24 h. A control group with fresh standard medium and positive and negative controls was included in the 96-well culture plate (eight wells for each test material). The experiments were conducted in triplicate, and cell viability was assessed following 24 h of incubation at 37 °C under 5% CO_2_.

#### 4.12.2. MTT Assay

The test materials were aspirated from each well of the initial plate. Subsequently, 25 μL of MTT reagent was pipetted into each well and incubated for 2 h at 37 °C in a CO_2_ incubator. Subsequently, the formazan crystals formed were solubilized using DMSO. The absorbance of the samples was then quantified at a wavelength of 540 nm using a Multi-Mode Microplate Reader Synergy HTX spectrophotometer (Agilent Technologies, Santa Clara, CA, USA). Finally, the cell viability was calculated according to Equation (4):

(4) $$CV(\%)=\frac{{OD}_{sample}-{OD}_{blank}}{{OD}_{control}-{OD}_{blank}}\times 100$$

where *CV*(%): cell viability; *OD*: optical density of the wells containing cells with the evaluated sample (*OD_sample_*), only cells (*OD_control_*), and cell culture media without cells (*OD_blank_*).

As per the producer’s specifications, the positive control consists of untreated cells, MTT solution, and DMSO, while the negative control consists of only dead cells, MTT solution, and DMSO. The IC_50_ values denote the concentrations (75, 100, 125, 150, 175, and 200 μg/mL) at which both samples (*H. sphondylium* and HS-Ag system) displayed 50% cell viability for NHDF and HeLa cell lines. The cell viability data were plotted on a graph, and the IC_50_ values were subsequently calculated [92].

### 4.13. Statistical Analysis

All experiments were performed in triplicate for all samples, all calibration curves, and concentrations. Statistical analysis was carried out using Student’s *t*-test and expressed as mean ± standard deviation (SD) using Microsoft Office Excel 2019 (Microsoft Corporation, Redmond, WA, USA). Dunnett’s multiple comparison post hoc test following a one-way analysis of variance test (ANOVA) was used to analyze the results. *p*-values <0.05 were considered statistically significant.

## 5. Conclusions

This study discusses the development of a novel plant-based system using AgNPs. FTIR, SEM, XRD, and DLS findings confirmed the successful incorporation of AgNPs into herbal matrix (*H. sphondylium*) particles and pores, resulting in the preparation of the HS-Ag system. Additionally, the antioxidant screening, antimicrobial, and in vitro cell viability investigations demonstrated that this innovative system exhibits enhanced biological properties compared to *H. sphondylium*. Collectively, this research work suggests that this new phytocarrier (HS-Ag system) holds promise for a wide range of medical applications.

## Figures and Tables

**Table 1:** Main phytochemicals identified by GC–MS analysis of *H. sphondylium* sample.

No.	RT [min]	RI Determined	Area [%]	Compound Name	Ref.
1	3.13	821	1.18	2-hexenal	[33]
2	5.73	1021	0.86	*p*-cymene	[34]
3	6.39	938	1.52	α-pinene	[34]
4	9.65	1228	0.67	cuminaldehyde	[35]
5	7.87	1034	1.48	limonene	[34,36]
6	11.60	1488	4.43	β-ionone	[34,36]
7	12.46	988	3.36	myristicin	[37]
8	16.15	1090	0.61	linalool	[34,36]
9	17.14	1212	1.56	myrtenal	[38]
10	18.32	1843	4.51	anethole	[34]
11	19.42	1165	0.79	decanal	[39]
12	20. 09	1473	19.49	α-curcumene	[34]
13	21.43	1247	1.85	carvone	[34,36]
14	22.67	1663	3.42	apiole	[40]
15	23.39	3113	1.08	campesterol	[41]
16	25.66	4776	4.81	*n*-hentriacontane	[42]
17	27.19	1365	4.76	vanillin	[39]
18	28.81	3333	11.78	β-amirin	[43]
19	30.68	1587	3.12	spathulenol	[34]
20	32.38	1193	0.37	octyl acetate	[44]
21	36.65	3139	0.92	stigmasterol	[45]
22	37.17	3289	4.38	β-sitosterol	[45]
23	37.57	1293	2.29	germacrene D	[34,46]
24	49.57	1507	0.89	cadinene	[46]
25	55.89	1627	2.25	cadinol	[46]

GC–MS: gas chromatography–mass spectrometry; RI: retention index (RIs calculated based upon a calibration curve of a C8–C20 alkane standard mixture); RT: retention time.

**Table 2:** Biomolecules identified by mass spectrometry analysis in *H. sphondylium* sample.

No.	Detected *m*/*z*	Theoretical *m*/*z*	Molecular Formula	Tentative of Identification	Category	Ref.
1	76.07	75.07	C_2_H_5_NO_2_	glycine	amino acids	[47]
2	90.88	89.09	C_3_H_7_NO_2_	alanine	amino acids	[47]
3	106.08	105.09	C_3_H_7_NO_3_	serine	amino acids	[47]
4	121.13	119.12	C_4_H_9_NO_3_	threonine	amino acids	[47]
5	134.11	133.10	C_4_H_7_NO_4_	aspartic acid	amino acids	[47]
6	148.12	147.13	C_5_H_9_NO_4_	glutamic acid	amino acids	[47]
7	187.15	186.16	C_11_H_6_O_3_	angelicin	coumarins	[1]
8	193.17	192.17	C_10_H_8_O_4_	scopoletin	coumarins	[1]
9	203.17	202.16	C_11_H_6_O_4_	xanthotoxol	coumarins	[16]
10	217.21	216.19	C_12_H_8_O_4_	sphondin	coumarins	[16]
11	247.22	246.21	C_13_H_10_O_5_	isopimpinellin	coumarins	[2]
12	271.29	270.28	C_16_H_14_O_4_	imperatorin	coumarins	[1,48]
13	287.27	286.28	C_16_H_14_O_5_	heraclenin	coumarins	[1,2,48]
14	305.28	304.29	C_16_H_16_O_6_	heraclenol	coumarins	[1,2,48]
15	317.31	316.30	C_17_H_16_O_6_	byakangelicol	coumarins	[48]
16	173.25	172.26	C_10_H_20_O_2_	capric acid	fatty acids	[1]
17	201.33	200.32	C_12_H_24_O_2_	lauric acid	fatty acids	[1]
18	229.37	228.37	C_14_H_28_O_2_	myristic acid	fatty acids	[15]
19	255.42	254.41	C_16_H_30_O_2_	palmitoleic acid	fatty acids	[15]
20	257.43	256.42	C_16_H_32_O_2_	palmitic acid	fatty acids	[1]
21	271.49	270.50	C_17_H_34_O_2_	margaric acid	fatty acids	[15]
22	281.39	280.40	C_18_H_32_O_2_	linoleic acid	fatty acids	[1,16]
23	283.51	282.50	C_18_H_34_O_2_	oleic acid	fatty acids	[1]
24	284.49	284.50	C_18_H_36_O_2_	stearic acid	fatty acids	[1]
25	313.49	312.50	C_20_H_40_O_2_	arachidic acid	fatty acids	[15]
26	341.59	340.60	C_22_H_44_O_2_	behenic acid	fatty acids	[15]
27	271.25	270.24	C_15_H_10_O_5_	apigenin	flavonoids	[8,10]
28	287.23	286.24	C_15_H_10_O_6_	kaempferol	flavonoids	[8,10]
29	291.28	290.27	C_15_H_14_O_6_	catechin	flavonoids	[10]
30	303.24	302.23	C_15_H_10_O_7_	quercetin	flavonoids	[8,10]
31	449.41	448.40	C_21_H_20_O_11_	astragalin	flavonoids	[1]
32	465.39	464.40	C_21_H_20_O_12_	hyperoside	flavonoids	[1]
33	611.49	610.50	C_27_H_30_O_16_	rutin	flavonoids	[8]
34	377.35	376.36	C_16_H_24_O_10_	loganic acid	iridoids	[1]
35	139.11	138.12	C_7_H_6_O_3_	*p*-hydroxybenzoic acid	phenolic acids	[10]
36	155.13	154.12	C_7_H_6_O_4_	gentisic acid	phenolic acids	[8]
37	165.15	164.16	C_9_H_8_O_3_	*p*-coumaric acid	phenolic acids	[8,10]
38	171.11	170.12	C_7_H_6_O_5_	gallic acid	phenolic acids	[10]
39	181.17	180.16	C_9_H_8_O_4_	caffeic acid	phenolic acids	[8,10]
40	195.18	194.18	C_10_H_10_O_4_	ferulic acid	phenolic acids	[8,10]
41	355.32	354.31	C_16_H_18_O_9_	chlorogenic acid	phenolic acids	[8]
42	149.19	148.20	C_10_H_12_O	estragole	phenylpropanoids	[49]
43	401.71	400.70	C_28_H_48_O	campesterol	sterols	[15]
44	413.69	412.70	C_29_H_48_O	stigmasterol	sterols	[15]
45	415.71	414.70	C_29_H_50_O	β-sitosterol	sterols	[1,15]
46	135.23	134.22	C_10_H_14_	*p*-cymene	terpenoids	[14,17]
47	137.24	136.23	C_10_H_16_	α-pinene	terpenoids	[14,17]
48	151.23	150.22	C_10_H_14_O	carvone	terpenoids	[49]
49	153.22	152.23	C_10_H_16_O	phellandral	terpenoids	[49]
50	155.25	154.25	C_10_H_18_O	linalool	terpenoids	[49]
51	156.25	156.26	C_10_H_20_O	menthol	terpenoids	[49]
52	193.31	192.30	C_13_H_20_O	β-ionone	terpenoids	[49]
53	203.34	202.33	C_15_H_22_	α-curcumene	terpenoids	[17,50]
54	205.36	204.35	C_15_H_24_	germacrene D	terpenoids	[14,17]
55	207.36	206.37	C_15_H_26_	cadinene	terpenoids	[51]
56	221.34	220.35	C_15_H_24_O	spathulenol	terpenoids	[50]
57	223.38	222.37	C_15_H_26_O	cadinol	terpenoids	[51]
58	251.34	250.33	C_15_H_22_O_3_	xanthoxin	terpenoids	[48]
59	273.51	272.50	C_20_H_32_	β-springene	terpenoids	[50]
60	427.69	426.70	C_30_H_50_O	β-amirin	terpenoids	[48]
61	149.21	148.20	C_10_H_12_O	anethole	miscellaneous	[1]
62	151.23	150.22	C_10_H_14_O	myrtenal	miscellaneous	[17]
63	153.16	152.15	C_8_H_8_O_3_	vanillin	miscellaneous	[10]
64	193.22	192.21	C_11_H_12_O_3_	myristicin	miscellaneous	[14]
65	223.25	222.24	C_12_H_14_O_4_	apiole	miscellaneous	[2]
66	255.23	254.24	C_15_H_10_O_4_	chrysophanol	miscellaneous	[1]
67	131.22	130.23	C_8_H_18_O	*n*-octanol	alcohols	[14]
68	117.19	116.20	C_7_H_16_O	heptanol	alcohols	[49]
69	75.13	74.12	C_4_H_10_O	butanol	alcohols	[49]
70	103.18	102.17	C_6_H_14_O	hexanol	alcohols	[49]
71	99.15	98.14	C_6_H_10_O	hexanal	aldehydes	[14,17]
72	129.22	128.21	C_8_H_16_O	octanal	aldehydes	[17]
73	157.25	156.26	C_10_H_20_O	decanal	aldehydes	[17]
74	145.22	144.21	C_8_H_16_O_2_	isobutyl isobutyrate	esters	[17]
75	163.19	162.18	C_10_H_10_O_2_	methyl cinnamate	esters	[1]
76	173.27	172.26	C_10_H_20_O_2_	octyl acetate	esters	[14]
77	187.28	186.29	C_11_H_22_O_2_	hexyl 2-methyl butanoate	esters	[17]
78	199.31	198.30	C_12_H_22_O_2_	dihydrolinalyl acetate	esters	[17]
79	197.28	196.29	C_12_H_20_O_2_	bornyl acetate	esters	[17]
80	201.33	200.32	C_12_H_24_O_2_	octyl isobutyrate	esters	[14,17]
81	229.36	228.37	C_14_H_28_O_2_	octyl hexanoate	esters	[14]
82	219.37	218.38	C_16_H_26_	5-phenyldecane	hydrocarbons	[46]
83	261.49	260.50	C_19_H_32_	4-phenyltridecane	hydrocarbons	[46]
84	353.69	352.70	C_25_H_52_	pentacosane	hydrocarbons	[1]
85	381.69	380.70	C_27_H_56_	heptacosane	hydrocarbons	[1]
86	395.81	394.80	C_28_H_58_	octacosane	hydrocarbons	[1]
87	423.79	422.80	C_30_H_62_	triacontane	hydrocarbons	[1]
88	437.81	436.80	C_31_H_64_	*n*-hentriacontane	hydrocarbons	[1]

**Table 3:** Volatile organic compounds identified via mass spectrometry in *H. sphondylium* sample.

Volatile Organic Compound	Odor Profile
*p*-cymene	woody
α-pinene	piney
carvone	minty
phellandral	pungent, terpenic
linalool	floral, woody
menthol	minty
β-ionone	woody
α-curcumene	herbal
germacrene D	woody
cadinene	woody
spathulenol	herbal, fruity
cadinol	herbal
xanthoxin	floral
anethole	minty
myrtenal	herbal
vanillin	vanilla
myristicin	spicy
apiole	herbal
hexanal	herbal
octyl acetate	fruity
octyl butyrate	fruity
octanal	citrus
decanal	citrus
isobutyl isobutyrate	sweet
methyl cinnamate	fruity
hexyl 2-methyl butanoate	sweet, fruity
bornyl acetate	piney
octyl hexanoate	fruity, herbal

**Table 4:** Characteristic absorption bands associated with phytoconstituents from *H. sphondylium* sample.

Biomolecules Category	Wavenumber [cm^−1^]	Ref.
terpenoids	2974, 2943, 2350, 1746, 1708, 1450, 1088, 882	[52]
coumarins	1730, 1630, 1608, 1589, 1565, 1510, 1265, 1140	[53]
flavonoids	4002–3124, 3402–3102, 1654, 1645, 1619, 1574, 1504, 1495, 1480, 1368, 1271, 1078, 768, 536	[54,55]
phenolic acids	3442, 1733, 1634, 1594, 1516, 1458, 1242, 1158, 881	[52,56]
amino acids	3400, 3332–3128, 2922, 2362, 2133, 1724–1755, 1689, 1677, 1649, 1644, 1643, 1632, 1628, 1608, 1498–1599	[52]
fatty acids	3606, 3009, 2962, 2932, 2848, 1700, 1349, 1249, 1091, 722	[36]
iridoids	1448, 1371, 1346, 1235, 1151	[57]
phytosterols	3431, 3028, 2938, 1641, 1463, 1060	[57,58]
phenylpropanoids	3188, 3002, 1636, 1504, 1449, 1248	[59]

**Table 5:** Antioxidant assays outcomes for both samples (*H. sphondylium* and HS-Ag system).

Sample	TPC [mg GAE/g]	FRAP [mM Fe^2+^]	DPPH IC_50_ [mg/mL]
*H. sphondylium*	8.14 ± 0.18	29.31 ± 0.11	7.65 ± 0.05
HS-Ag system	11.47 ± 0.16	32.44 ± 0.08	5.62 ± 0.07

Values are expressed as the mean ± SD (*n* = 3). DPPH: 2,2-Diphenyl-1-picrylhydrazyl; FRAP: ferric reducing antioxidant power; GAE: gallic acid equivalents; IC_50_: half maximal inhibitory concentration; TPC: total phenolic content.

**Table 6:** Results of antibacterial activity against selected pathogenic microorganisms.

Pathogenic Microorganism	Sample	Inhibition Zone Diameter [mm]						
		Sample Concentration [μg/mL]					Positive Control (Gentamicin 100 μg/mL)	Negative Control (DMSO)
		100	125	150	175	200		
*Staphylococcus* *aureus*	*H. sphondylium*	11.23 ± 0.75	13.98 ± 1.17	17.06 ± 0.68	21.19 ± 0.72	25.46 ± 0.45	9.57 ± 0.35	0
	citrate-coated AgNPs	13.03 ± 0.51	16.45 ± 0.55	18.85 ± 0.48	26.94 ± 0.62	30.13 ± 0.42		
	HS-Ag system	14.78 ± 0.54	17.27 ± 0.78	21.62 ± 0.47	28.52 ± 0.56	34.14 ± 0.56		
*Bacillus subtilis*	*H. sphondylium*	19.83 ± 0.09	21.47 ± 0.43	24.36 ± 0.32	27.69 ± 0.38	31.22 ± 0.31	17.89 ± 0.28	0
	citrate-coated AgNPs	21.32 ± 0.31	24.76 ± 0.27	26.74 ± 0.19	30.23 ± 0.22	34.58 ± 0.24		
	HS-Ag system	23.11 ± 0.41	25.38 ± 0.36	29.51 ± 0.16	32.76 ± 0.47	36.25 ± 0.28		
*Pseudomonas* *aeruginosa*	*H. sphondylium*	10.64 ± 0.27	14.09 ± 0.21	16.73 ± 0.25	18.95 ± 0.82	20.38 ± 0.17	18.67 ± 0.19	0
	citrate-coated AgNPs	9.84 ± 0.19	11.72 ± 0.23	13.81 ± 0.34	16.45 ± 0.42	18.52 ± 0.17		
	HS-Ag system	21.78 ± 0.19	23.01 ± 0.17	24.74 ± 0.32	26.18 ± 0.61	27.65 ± 0.19		
*Escherichia coli*	*H. sphondylium*	11.84 ± 0.37	14.69 ± 0.34	17.15 ± 0.51	19.03 ± 0.43	21.49 ± 0.34	20.69 ± 0.31	0
	citrate-coated AgNPs	13.12 ± 0.21	17.26 ± 0.27	20.07 ± 0.33	22.21 ± 0.45	25.89 ± 0.42		
	HS-Ag system	20.88 ± 0.28	21.63 ± 0.25	23.06 ± 0.42	25.02 ± 0.47	27.12 ± 0.58		

Values are expressed as the mean ± SD (*n* = 3). DMSO: Dimethyl sulfoxide; HS-Ag: *H. sphondylium*–silver nanoparticle (AgNP) system; SD: standard deviation.

**Table 7:** MICs and MBCs of samples against selected pathogenic microorganisms.

Pathogenic Microorganism	Sample	MIC [μg/mL]	MBC [μg/mL]	Gentamicin	
				MIC [μg/mL]	MBC [μg/mL]
*Staphylococcus aureus*	*H. sphondylium*	0.22 ± 0.07	0.23 ± 0.19	0.62 ± 0.22	0.62 ± 0.21
	citrate-coated AgNPs	0.14 ± 0.05	0.13 ± 0.04		
	HS-Ag system	0.12 ± 0.03	0.11 ± 0.16		
*Bacillus subtilis*	*H. sphondylium*	0.28 ± 0.19	0.24 ± 0.12	0.49 ± 0.18	0.43 ± 0.19
	citrate-coated AgNPs	0.18 ± 0.12	0.19 ± 0.08		
	HS-Ag system	0.16 ± 0.08	0.15 ± 0.23		
*Pseudomonas aeruginosa*	*H. sphondylium*	0.98 ± 0.11	0.99 ± 0.14	1.27 ± 0.16	1.26 ± 0.19
	citrate-coated AgNPs	0.67 ± 0.21	0.67 ± 0.17		
	HS-Ag system	0.52 ± 0.07	0.59 ± 0.37		
*Escherichia coli*	*H. sphondylium*	0.38 ± 0.09	0.31 ± 0.21	0.82 ± 0.19	0.82 ± 0.17
	citrate-coated AgNPs	0.30 ± 0.08	0.31 ± 0.11		
	HS-Ag system	0.26 ± 0.13	0.26 ± 0.15		

Values are expressed as the mean ± SD (*n* = 3). HS-Ag: *H. sphondylium*–silver nanoparticle (AgNP) system; MBC: minimum bactericidal concentration; MIC: minimum inhibitory concentration; SD: standard deviation.

## Data Availability

The original data presented in the study are openly available in [GoFile repository] at [https://gofile.me/7rkqY/KHgZHOglD, accessed on 20 August 2024].
